# The Impact of Green Tea and Its Bioactive Compounds on Mood Disorder Symptomology and Brain Derived Neurotrophic Factor: A Systematic Review of Randomized Controlled Trials

**DOI:** 10.3390/biomedicines13071656

**Published:** 2025-07-07

**Authors:** Aidan M. Cavanah, Laura A. Robinson, Madison L. Mattingly, Andrew D. Frugé

**Affiliations:** 1 College of Nursing, Auburn University, 710 S. Donahue Drive, Auburn, AL 36849, USA; lad0030@auburn.edu (L.A.R.); adf0003@auburn.edu (A.D.F.); 2 Department of Nutritional Sciences, Auburn University, 210 Spidle Hall, Auburn, AL 36849, USA; 3 School of Kinesiology, Auburn University, 301 Wire Road, Auburn, AL 36849, USA; mlm0139@auburn.edu

**Keywords:** green tea, depression, anxiety, stress, brain-derived neurotrophic factor (BDNF), catechins

## Abstract

**Background/Objectives**: Mood disorders include symptoms of depression, anxiety, and or stress, and have increased in prevalence. Green tea and its bioactive components (epigallocatechin gallate [EGCG] and L-theanine) have been investigated for their health benefits and neuroprotective properties. As adults seek integrative and alternative treatment modalities, it is relevant to determine the effects of natural and non-pharmacological treatments on humans. This study aimed to assess current evidence from published randomized controlled trials testing the effects of green tea, green tea extracts, or its bioactive compounds on mood disorder symptomology and brain-derived neurotrophic factor (BDNF). **Methods**: We searched PubMed, Cochrane Library, PsycINFO, Embase, Google Scholar, and ClinicalTrials.gov, following the Preferred Reporting Items for Systematic Reviews and Meta-Analyses (PRISMA) checklist and utilizing predetermined inclusion and exclusion criteria. **Results**: A total of 445 studies were identified, 395 screened, and thirteen met inclusion criteria. Seven used one of the bioactive compounds found in green tea for intervention, while six used green tea extract, matcha, or traditional green tea. Mood disturbance was assessed with several tools, with studies reporting improvements in depressive (n = 4), anxiety (n = 6), stress (n = 5), and sleep (n = 1) symptoms. No studies found a statistically significant effect of green tea or its bioactive compounds on BDNF. Conclusions: Our findings suggest green tea, GTE, L-theanine, and EGCG may improve mood disorder symptomology, particularly symptoms of depression; no evidence to date reports effects on BDNF.

## 1. Introduction

Mood is defined as an internal, sustained feeling that impacts an individual’s behaviour, emotional state, and interaction with the external world [1]. According to the Diagnostic and Statistical Manual of Mental Disorders, Fifth Edition (DSM-5), mood disorders fall under two broad categories, bipolar and depressive disorders, with anxiety disorders being considered differential diagnoses [2]. Most recently, the prevalence of mood disorders was more than 40% in the “emerging adulthood” population, spanning 18–29 years of age [3]. According to the Centers for Disease Control (CDC), the percentage of adults who received mental health treatment over the previous 12 months increased from 19.2% to 23.9% from 2019 to 2023 [4]. In the world of mental health and mood disorders, specifically depression, pharmacological intervention is standard. This has been proven to be somewhat problematic, as antidepressants have been found to be less useful in cases of mild to moderate depression, which constitutes the majority of individuals with depression [5]. Pharmacological interventions continue to relieve symptoms but lack resolutions relating to the underlying condition [6].

In addition to pharmacological treatment, psychotherapy, such as cognitive behavioural therapy (CBT), is a well-established and effective treatment for mood disorders [7]. These approaches aim to identify and mitigate maladaptive cognitive thoughts and events that affect behaviour, with the goal of achieving desired behaviour change [8]. While this approach is effective, barriers such as accessibility, stigma, and cost have led individuals to seek out alternative methods of treatment [9]. Increasing interest in alternative treatments like plants and their constituents as natural remedies warrants further investigation.

One alternative treatment that is already popular, being the second most consumed beverage in the world, only behind water, comes from the *Camellia* plant and is commonly known as tea [10]. Most teas come from the *Camellia sinensis* plant and are classified largely based on antioxidant and fermentation level, with green being unfermented, oolong semi-fermented, and black fully fermented [11]. Green tea has the highest concentration of catechins, a group of bioactive polyphenols [12]. Of these catechins, epigallocatechin-3-gallate (EGCG) makes up around a third of the catechin weight alone [13]. EGCG has been found to bind to cell surface receptors like 67-kDa laminin receptor (anti-tumor) and toll-like receptor (anti-inflammatory) [14], with other published studies reporting it has antioxidant, anticancer, antidiabetic, antimicrobial, and neuroprotective roles [15]. Regarding its neuroprotective effects, research suggests EGCG inhibits oxidative injury by preventing pro-oxidative enzymes and acting as a potent antioxidant, thus preventing neuroinflammation and potential neurotransmitter dysfunction [16]. Isolated EGCG isn’t the only potent green tea supplement with promising research. Green tea extracts, which contain high concentrations of EGCG, have been found to improve rodent brain dysfunction through anti-oxidative measures after a high-fat diet intervention. The authors reported that the mice that consumed GTE had significantly higher memory retention, a significant increase in total brain weight, and a significant decrease in the accumulation of Aβ 1–42 in the cerebral cortex (all *p* < 0.05) [17]. High-temperature processed green tea extract was found to prevent cognitive impairments and restore synaptic plasticity related to post-menopausal depression [18]. Overall, the accumulating evidence suggests that GTE possesses potent neurobiological properties, making it a compelling focus for nutritional neuroscience research.

Another highly active compound found in green tea and its extracts is L-theanine. L-theanine is an amino acid found in tea that modulates neurological functions associated with stress, mood, and sleep. Mechanistically, L-theanine delays neuronal death, reduces glutamate excitotoxicity, and can promote neurogenesis [19]. This interaction with glutamate is due to L-theanine’s structure resembling glutamate, which allows it to compete for glutamate receptors [20]. Along with glutamate, studies suggest L-theanine increases gamma-aminobutyric acid (GABA), crucial for the promotion of stress reduction and relaxation, as well as modulation of serotonin and dopamine [21]. An open-label clinical trial found that 250 mg of L-theanine over 8-weeks reduced Hamilton Depression Rating Scale scores (*p* = 0.007), trait anxiety scores (*p* = 0.012) and Pittsburgh Sleep Quality Index scores (*p* = 0.030) in patients with major depressive disorder [22] L-theanine crosses the blood brain barrier by ways of the leucine-preferring transport system, directly affecting brain metabolism [23]. Another 8-week study in schizophrenic patients found 400 mg/d of L-theanine to reduce anxiety symptoms and dysphoric mood, coupled with antipsychotic therapy [24].

The discovery of how these compounds act on neurobiology has led to a discussion surrounding BDNF. BDNF is a neurotrophin that modulates brain plasticity, with research showing its effects on psychiatric disorders [25]. BDNF has been found in high concentrations in the hippocampus and frontal cortex, where it exerts neuroprotective and neuroactivating properties on excitatory and inhibitory neurons [26]. Serum BDNF is a potential biomarker of psychiatric illness and is reflective of brain-tissue BDNF levels [27], with increases in BDNF associated with improved depressive symptoms [28]. These studies support the ‘neurotrophin hypothesis of depression’, which states reduced levels of BDNF in the brain contribute to cell atrophy in the hippocampus and prefrontal cortex [29]. This hypothesis goes further by implying neurogenesis impairment is a response to decreased BDNF expression. Thus, antidepressants may be effective due to their ability to increase the expression of BDNF, improving neuronal plasticity [30].

Beyond its direct effects, studies have found that BDNF exhibits a level of neuromodulation on monoamines, with one rat study reporting that BDNF infusion into the midbrain increased serotonin, dopamine, and norepinephrine pathway activity in multiple forebrain areas [31]. A key component to the success of BDNF is the transmembrane receptor TrkB (tropomyosin receptor kinase B), which has been found to mediate the action of BDNF [32]. Other complementary approaches using flavonoids have found that they can activate TrkB and provide BDNF-dependent neuronal protection [33]. Signaling of BDNF through this receptor has been found to be necessary for antidepressant effects on behavior [34]. Mouse models have allowed researchers to investigate this effective partnership of BDNF and TrkB. Deletion of BDNF from the dentate gyrus cells of mice and TrkB from progenitor cells of dentate granule neurons both saw preventative effects of antidepressants [35]. Together, these findings highlight the critical role of BDNF-TrkB signaling in mediating antidepressant effects and suggest that dietary compounds capable of modulating this pathway, such as green tea, warrant further investigation in the treatment of mood disorders.

Given this background, the aim of this study is to evaluate the effects of green tea and its bioactive compounds—specifically L-theanine and epigallocatechin gallate (EGCG)—on mood disorder symptomology and BDNF levels using evidence from published randomized controlled trials. From this aim, we hypothesized that green tea and its bioactive compounds would improve mood disorder symptomology and increase BDNF levels in randomized controlled trials

## 2. Materials and Methods

### 2.1. Protocol Registration

This systematic review was prospectively registered on 11 July 2024 with PROSPERO, study ID: CRD42024559701, available from: https://www.crd.york.ac.uk/prospero/display_record.php?ID=CRD42024559701, accessed on 11 July 2024. Using the following databases PubMed, Cochrane Library, PsycINFO, Embase, Google Scholar and ClinicalTrials.gov, from index dates between June and September of 2024, we conducted a systematic review of randomized controlled trials to evaluate the effects of green tea consumption on various mood disorders like depression, anxiety, stress, sleep and bipolar disorder. This study was exclusively performed by the present investigators and was conducted in accordance with the 2020 Preferred Reporting Items for Systematic Reviews and Meta-Analyses (PRISMA) statement [36]; the completed checklist is available in the Supplementary Materials.

### 2.2. Search Strategy and Eligibility Criteria

Studies published were retrieved from the PubMed, Cochrane Library, and Embase electronic databases by the author, AC. A search strategy was designed utilizing the following keywords, “mood disorders,” “depression,” anxiety,” “bipolar disorder” and were paired with the following words, “green tea,” epigallocatechin gallate,” “catechins, “EGCG,” “brain derived neurotrophic factor,” or BDNF.” Boolean operators were applied to structure the search as follows: (“green tea” OR “EGCG” OR “epigallocatechin gallate” OR “catechins” OR “L-theanine”) AND (“depression” OR “mood disorders” OR “anxiety” OR “bipolar disorder”) AND (“BDNF” OR “brain-derived neurotrophic factor”) AND (“randomized controlled trial” OR “RCT”). We did not contact any of the authors of the studies that were extracted since a meta-analysis was not performed.

### 2.3. Study Selection

The inclusion criteria (Table 1) were randomized controlled trials that (a) investigated the impact of green tea or one of its bioactive compounds on mood disorder symptomology or BDNF levels as its primary outcome; (b) were published in the English language; (c) had a sample population of adults over the age of 18; (d) included a sample population of healthy adults or those with elevated levels or a diagnosis of mood disorders, including but not limited to, depression, anxiety, bipolar, stress and sleep disorders at the study screening. We excluded (e) sample populations with existing hormone imbalance conditions (i.e., pregnant, nursing, menopause); (f) studies utilizing multicomponent dietary/supplement interventions; (g) randomized controlled trials without a placebo, control or no intervention group; (h) any epidemiological, observational, longitudinal or cross-sectional studies. One author (AC) conducted the search for related studies; two authors (AC and LR) determined whether studies met inclusion criteria, with discrepancies resolved by a third author (MM).

### 2.4. Data Extraction

All data were extracted using the Covidence data extraction tool, a web-based platform designed to facilitate study screening, data extraction, and quality assessment of systematic reviews. The following study information was gathered, Identification (Country of origin, first author’s name and institution), Methods (study design, duration of intervention, participant demographics), Population (inclusion and exclusion criteria, group differences, sample size, withdrawals and baseline characteristics), Intervention (allocation, dose and frequency), Outcomes (scales used to measure outcomes, timepoints, scale direction and data values) and Results (mean and standard deviation values). The extraction process was guided by a customized template within Covidence, and any discrepancies in data extracted between reviewers were resolved through discussion or consensus by a third reviewer.

### 2.5. Risk of Bias Tools

**Figure 1:**
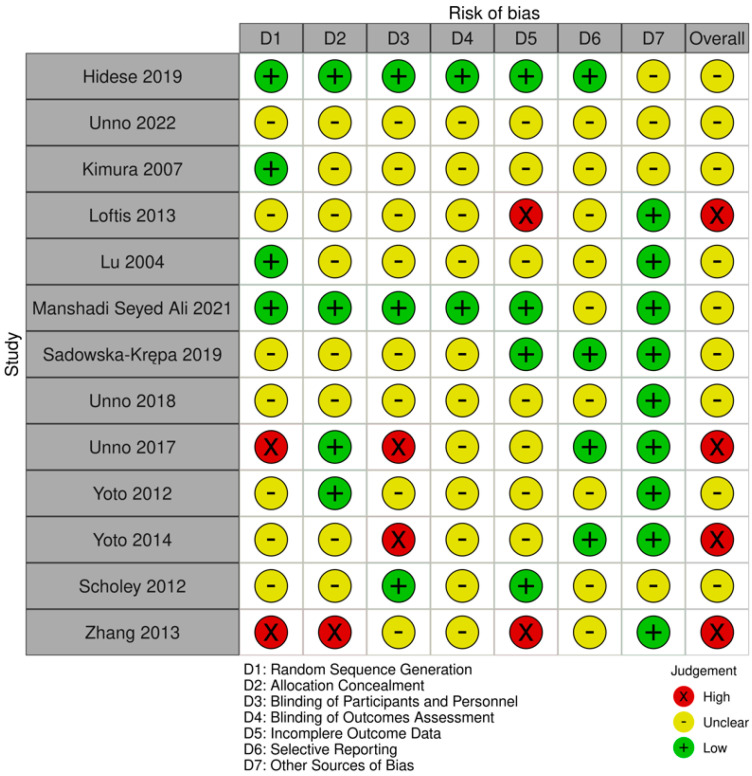
Risk of bias assessments and methodological quality for each study included, using the Robvis tool [37,38,39,40,41,42,43,44,45,46,47,48,49].

Risk of bias for the included studies was assessed using the Cochrane Risk of Bias tool [37]. Two reviewers independently (AC and LR) assessed the risk of bias for each study. Discrepancies were resolved with discussion, and a third reviewer (MM) was consulted if consensus could not be reached. The Cochrane guidelines to assess risk of bias used the following seven domains: random sequence generation, allocation concealment, blinding of participants and personnel, blinding of outcome assessment, incomplete outcome data, selective reporting bias, and other potential sources of bias. Based on these domains, the studies were provided a score of “low”,” high,” or “unclear.” The risk of bias summary figure was generated using the robvis-tool “traffic light” plot (see Figure 1). Upon selection, there were no discrepancies among the selected studies between the authors (AC and LR) in determining study eligibility. Inter-rater agreement was assessed using Cohen’s kappa, calculated in SPSS v29.0 (IBM Corp, Armonk, NY, USA), yielding a value of κ = 1.00, indicating perfect agreement.

## 3. Results

### 3.1. Search Results

**Figure 2:**
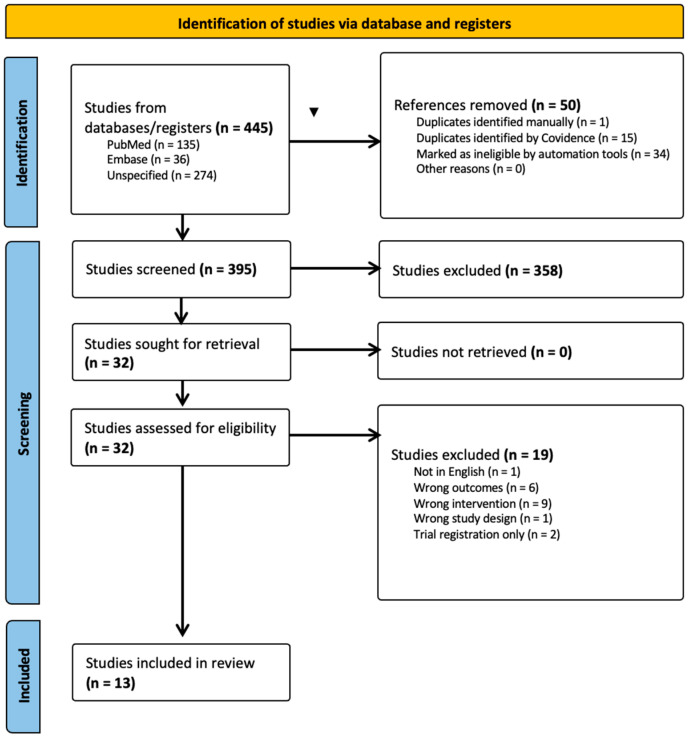
PRISMA 2020 Flow diagram for new systematic reviews including searches of databases and registers.

Initial search results identified 395 studies. After removing duplicates (*n* = 16), reviews, pilot studies, and articles not written in English, the number of full-text studies assessed for eligibility was reduced to 32. At full-text review, 19 studies were excluded due to various reasons: 9 using the wrong intervention, 6 having the wrong outcomes, 2 at trial registration only, 1 using the wrong study design, and 1 not in English. Thus, leaving 13 that met the eligibility criteria (see Figure 2).

### 3.2. Study Characteristics

Characteristics of the selected studies are listed in Table 2. The oldest study that met inclusion criteria was published in 2004, with the most recent being in 2022. Countries of origin details for the included studies were Japan, Poland, Australia, China, Iran, and the USA. All studies were randomized controlled trials with mood disturbance being measured with the Self-Rating Depression Scale, State-Trait Anxiety Inventory, Pittsburgh Sleep Quality Index, Hamilton Depression Rating Scale, Beck Depression Inventory-II, Beck Anxiety Inventory, and Profile of Mood States.

Participant ages ranged from 20 to 69 years. The proportion of male participants compared to females was 25% to 100%. The majority of the studies included healthy adults, with the exception of [38] those [39] that included men and women with psychiatric or depressive conditions. Sample sizes ranged from 12 to 81 participants, with two studies comprising only males. Intervention types included green tea extract (GTE), matcha, L-theanine, and EGCG, with dosages ranging from 150 mg to 1.2 g/day. Intervention durations varied from a single session to 12 weeks, though the most common time frame was 2 to 6 weeks. The most commonly investigated bioactive compound was L-theanine, which consistently demonstrated reductions in perceived stress and anxiety, especially under acute cognitive or stress-inducing tasks. Studies examining GTE or EGCG had some trials reporting improvements in depressive symptoms and others finding no significant differences when compared to placebo. Only one study reported on BDNF concentrations, noting slight increases in both groups, most likely due to exercise rather than the supplement itself. A complete summary of the study populations, sample sizes, intervention characteristics, outcome measures, and results is presented in Table 2.

### 3.3. Study Interventions and Dosages

Interventions included green tea extracts, L-theanine, and EGCG administered in doses ranging from 200 mg to 400 mg per day. Intervention durations varied from single-session studies, e.g., [44,45,46,47] to 12 weeks [49].

### 3.4. Outcome Measures

Mood outcomes were assessed using validated scales, such as the State-Trait Anxiety Inventory (STAI), Profile of Mood States (POMS), and Hamilton Rating Scale for Depression (HDRS). Subjective stress levels were also measured using visual analog scales (VAS). SDS, PANSS, HAM-A, MADRS. Sleep was measured with PSQI.

#### 3.4.1. Anxiety

Eight studies examining green tea, isolated prominent compounds, and have found varying ratios to be notable. The sum of caffeine (C) and epigallocatechin gallate (E) relative to L-theanine (T) and arginine (A) was found to have significant implications for anxiety reduction. Two powdered green tea groups containing CE/TA ratios of 4.7 and 3.9, respectively, found the 3.9 CE/TA ratio of Group B to significantly decrease anxiety scores over a two-week intervention [38]. L-theanine reduced tension anxiety scores compared to placebo [40] (*p* = 0.004), and matcha tea significantly lowered state anxiety [44] (*p* = 0.03). A 2004 study found that a 200 mg dose of L-theanine significantly reduced subjective anxiety, indicated by the tranquil-troubled subscale score of the visual analog mood scale (VAMS), in a resting state. This was compared to alprazolam and placebo (*p* < 0.05) [43]. Participants who consumed a matcha with a higher L-theanine content (3 g/day) reported significantly lower STAI anxiety scores in the first 7 days compared to placebo (*p* = 0.03) [46].

#### 3.4.2. Depression

The longest study found a 12-week 400 mg GTE intervention improved depressive symptoms when examining HDRS scores compared to placebo (*p* = 0.035) in a population of people living with HIV [49]. Similarly, [46] reported significant reductions in MADRS (*p* < 0.05) and HRSD-17 (*p* < 0.001) scores from 46 participants following the 5-week green tea consumption of 400 mg 3x/day. One study reported supplementation with 150 mg of EGCG and placebo both improved psychotic, depressive, and anxiety symptoms (PANSS, *p* < 0.0001, HAM-A, *p* = 0.0032, HAM-D, *p* = 0.0003) with no significant differences between groups of patients with psychiatric diagnoses [39].

**Table 2:** Characteristics and findings of included studies evaluating green tea components on mood outcomes.

Author and Year	Study Population	Sample Size All (M; F)	Age (y) (mean ± SD)	Intervention, Dose	Duration of Intervention	Mood Outcome Measure	Result
Unno 2022 [40]	Healthy adults	81 (18 M; 63 F)	52.3 ± 15.7 (Group A), 54.5 ± 15.4 (Group B)	Green tea (3.9–4.7 CE/TA ratio), 3 × 1.5 g/day	2 weeks	Japanese STAI Form X-1, SDS	Group B: Significant decrease in STAI; depressive scores improved in both groups.
Unno 2017 [41]	5th year pharmaceutical science students	20 (10 M; 10 F)	23.2 ± 0.6 (Low caffeine), 22.4 ± 0.2 (Placebo)	Low-caffeine green tea (714 ± 79 mL/day), ~15 mg theanine/day	17 days	Japanese STAI Form X-1, VAS for stress	Low-caffeine group showed significantly lower stress (VAS) and reduced post-stress salivary α-amylase activity.
Sadowska 2019 [42]	Cross-Fit Trained Physical Education Faculty	16 (16 M)	22.0 ± 1.1 (GTE), 23.1 ± 1.7 (Placebo)	Green tea extract capsules (250 mg, 245 mg polyphenols/day)	6 weeks	BDNF levels	No marked differences; BDNF increased slightly after exercise in both groups.
Yoto 2014 [43]	Healthy adults	18 (9 M; 9 F)	23.4 ± 1.85	Shaded white tea (192 mg/caffeine, 223 mg/catechins) or *Sagara* green tea (87 mg/caffeine, 304 mg/catechins), or warm water	Single session, 3 trials over 3 weeks	POMS, TMD scores	Shaded white tea significantly reduced TMD scores of POMS and improved task performance compared to warm water.
Lu 2004 [44]	Healthy adults	16 (12 M; 4 F)	24.8 ± 5.4 (M), 29.0 ± 1.4 (F)	L-theanine (200 mg), alprazolam (1 mg) or placebo	3 days over 3 weeks	BAI, STAI, VAMS	L-theanine significantly reduced VAMS tranquil-troubled subscale score compared to placebo in relaxed condition.
Unno 2018 [45]	Healthy university students	39 (23 M; 16 F)	23 ± 1.1	Matcha tea (3 g/day) or placebo-matcha	15 days	Japanese STAI Form X-1	Matcha significantly reduced anxiety compared to placebo.
Yoto 2012 [46]	Healthy adults	16 (8 M; 8 F)	22.8 ± 2.1	L-theanine (200 mg) + placebo, caffeine (100 mg) + placebo or placebo	Single session, 3 trials over 3 weeks	POMS, VAS	L-theanine significantly reduced tension-anxiety scores compared to placebo.
Zhang 2013 [47]	Healthy adults	74 (46 completed: 23 M; 23 F)	25.7 ± 4.7	Green tea powder (400 mg, 3x/day) or cellulose placebo	5 weeks	MADRS, HRSD-17	Green tea significantly improved depressive symptoms (MADRS and HRSD-17 scores) compared to placebo.
Kimura 2007 [48]	Healthy Undergraduate students	12 (12 M)	21.5 ± 1.38	L-theanine (200 mg, 2 conditions) or placebo	Single session, 4 trials	STAI, VAS	L-theanine reduced stress (VAS) and state anxiety (STAI).
Loftis 2013 [38]	DSM-4-TR Axis I Diagnosis of schizophrenia, schizoaffective or bipolar disorder	34 (25 completed)	18 years or older	EGCG capsules (150 mg) or cellulose placebo capsules	8 weeks	HAM-D, HAM-A, PANSS	EGCG did not significantly affect psychiatric symptoms compared to placebo due to both groups seeing significant decreases.
Seyed Ali 2021 [39]	People with HIV undergoing antiretroviral therapy with diagnosed mild to moderate depression	50 (32 M; 18 F)	18–65 years	Green tea extract capsules (400 mg, 2x/day) or placebo	12 weeks	HDRS	GTE significantly improved (HDRS) depressive symptoms compared to placebo.
Hidese 2019 [49]	Healthy adults	30 (9 M; 21 F)	48.3 ± 11.9	L-theanine (200 mg/day) or placebo	4 weeks (crossover)	SDS, STAI, PSQI	L-theanine improved depression, anxiety, and sleep quality compared to placebo.
Scholey 2012 [50]	Healthy adults	31 (12 M; 19 F)	27.7 ± 9.3	EGCG (300 mg, caffeine-free) or placebo	Single session, 2 trials	Bond-Lader mood scale	EGCG increased calmness and reduced stress compared to placebo.

#### 3.4.3. Stress

In 19 participants consuming low-caffeine green tea containing ~15 mg of theanine and 3.6 mg of arginine daily, subjective stress levels measured with VAS were significantly lower compared to placebo (*p* = 0.0003) [41]. Shaded white tea (192 mg caffeine, 223 mg catechins) significantly reduced total mood disturbance (*p* < 0.01) after three mental stress load tasks compared to *Sagara* (87 mg caffeine, 304 mg catechins), which also saw reductions in TMD scores but to a lesser degree (*p* < 0.1) [42]. Oral intake of 200 mg L-theanine acutely lowered POMS tension-anxiety scores compared to placebo (*p* = 0.004) in response to psychological stressors [40]. A study performed in twelve male undergraduates taking a 200 mg L-theanine supplementation before and during a mental arithmetic task showed a significant reduction in their VAS perception of stress and their STAI state of anxiety compared to placebo [48]. The only study to use the Bond-Lader Mood scale found that 300 mg of EGCG significantly increased self-reported calmness (*p* = 0.04) and reduced self-reported stress (*p* = 0.017) following acute neurocognitive assessments [47].

#### 3.4.4. Sleep

One study to include sleep disturbance found 200 mg of L-theanine for 4-weeks showed significant improvements in depression (SDS, *p* = 0.019), anxiety (STAI, *p* = 0.006), and sleep quality (PSQI, *p* = 0.013) with specific improvements in sleep latency (*p* = 0.036) and daytime dysfunction (*p* = 0.022) compared to baseline. When compared to placebo, the L-theanine group showed significant improvements in PSQI subscale scores for sleep latency (*p* = 0.0499), sleep disturbance (*p* = 0.046), and use of sleep medication (*p* = 0.047), suggesting L-theanine has the potential to reduce mood disturbance and improve sleep quality in stressed individuals [50].

#### 3.4.5. BDNF

The only study to examine BDNF [44] had sixteen CrossFit males randomized to two groups, one consuming a placebo and one consuming 2 capsules of GTE, containing 200 mg catechins and 137 mg EGCG for six weeks. BDNF levels were comparable at baseline (*p* > 0.05), with post-intervention BDNF levels increasing by ~11% and 12% in placebo and GTE groups, respectively. With the GTE groups, there was no statistical significance compared to placebo (*p* > 0.05 for group x time interaction. Immediately post-exercise and 1-h post-recovery saw both groups having small increases in BDNF compared to resting levels (*p* < 0.001) but no significant differences between the groups (*p* > 0.05). The authors note that this slight increase in BDNF is most likely attributed to the exercise rather than supplementation.

### 3.5. Risk of Bias Assessment

Across the included studies, 31% had low risk of bias for random sequence generation, allocation concealment, incomplete outcome data, and selection reporting, 23% had low risk of bias for blinding of participants and personnel, 15% had low risk of bias for blinding of outcomes assessment and 69% had low risk of bias for other sources of bias. Of the studies included, 31% had an overall high risk of bias, with at least one of the seven domains showing high risk, 69 69% had an overall unclear risk of bias, given that at least one domain showed unclear risk of bias (see Figure 1).

## 4. Discussion

This systematic review evaluated the effects of green tea and its bioactive compounds (L-theanine, EGCG, etc.) on mood disturbance, including depression, anxiety, stress, and sleep, as well as BDNF. Thirteen randomized controlled trials (RCTs) were included, with dates of publication ranging from 2004 to 2022. The studies were conducted in various countries, including Japan, Poland, Australia, China, Iran, and the United States, involving participants aged 20 to 69 years. Most (*n* = 11) studies used healthy adults, with only two having participants with psychiatric or depressive conditions.

### 4.1. L-Theanine

L-theanine consistently reduced anxiety across multiple studies. This amino acid was found to reduce tension anxiety scores when compared to a placebo, as well as subjective anxiety in the tranquil-troubled subscale of a VAMS. In other L-theanine interventions, this compound was found to reduce stress perception and state anxiety under acute mental arithmetic tasks when compared to a placebo. Rat studies have outlined a potential mechanism for this by noting L-theanine’s ability to decrease adrenocorticotropic and corticosterone hormone levels in the hippocampus and prefrontal cortex [51]. Further animal studies have found suppression of adrenal hypertrophy and brain inflammation in mice that were provided a CE/TA (caffeine, EGCG, L-theanine, arginine) ratio of 2 to 8, as well as maintenance of *Npas*4, typically reduced in depression and anxiety, at a CE/TA ratio of 4 [7,43]. The ratio in which L-theanine was presented has proven to be important, with a powdered green tea ratio of CE/TA at 3.9 significantly decreasing anxiety scores over two weeks. Matcha with a higher theanine content was shown to lower STAI scores. These findings are supported by current studies, with one clinical trial in 2019 finding improvements in depression, anxiety, and sleep after a 200 mg/day dose of L-theanine over a 4-week period in patients with major psychiatric illness [22]. Rodent studies utilizing L-theanine from matcha found supporting results highlighting a reduction in stress-induced adrenal hypertrophy with L-theanine levels at 0.32 mg/kg or more [45]. L-theanine over four weeks was also found to improve sleep quality, specifically related to sleep latency and daytime dysfunction, compared to baseline and placebo. This finding suggests L-theanine could be beneficial in improving the sleep quality of stressed individuals. Based on the current published literature, it would be safe to say that doses of L-theanine ranging from 200 mg to 400 mg seem to have positive effects on mood disturbance symptomology, ranging from depression and anxiety to sleep. The findings of this systematic review with regard to L-theanine are consistent with other published reviews that find L-theanine supplementation to reduce psychiatric symptoms compared to controls [21]. This amino acid even has implications for inpatient treatment as well, with one study citing adjunctive L-theanine (400 mg/day) with risperidone in chronic schizophrenic patients found improvements in PANSS scores over an 8-week intervention [52]. The data shows promising applications for this compound in both inpatient and outpatient interventions for mental health disorders.

### 4.2. Green Tea Extracts

Green tea extracts showed promising results with their supplementation improving depressive symptoms, particularly in a population of depressed individuals when looking at HDRS scores [49]. Further supplementation with green tea saw reductions in MADRS scores as well. An interesting study utilizing participants with diagnosed psychiatric disorders found EGCG to improve PANSS, HAM-A, and HAM-D scores, ranging from anxiety, depressive, and psychotic symptoms. These findings are consistent with current reviews noting green tea and its neuroprotective effects, but more human trials are needed, as many studies utilize mouse models [53]. In human trials, several studies demonstrated that green tea and its bioactive compounds could improve subjective stress levels. EGCG was found to increase subjective calmness while reducing self-reported stress following acute neurocognitive assessments. Low caffeine green tea was found to reduce subjective stress levels when compared to a placebo. Shaded white tea specifically was found to significantly reduce total mood disturbance when compared to another green tea variant known as *Sagara*. This lends to the idea of varying types of green tea showing differing results that could benefit from further research. Given the interventions found for this review, it would be beneficial to compare two types of supplements, GTE and EGCG, to determine which has a greater impact on mood disturbance.

### 4.3. Green Tea and BDNF

Only one study was found to assess changes in BDNF levels, which found that both the GTE and placebo groups saw increases in BDNF but no difference between groups. The authors attribute this increase in BDNF over the course of the trial to physiological responses to the exercise, more so than the intervention. The importance of BDNF and its implications for mental health have been studied, with reports of lower serum BDNF in patients with major depressive disorder [54,55]. Interestingly, antidepressants have been found to increase serum BDNF [56]. Given the aforementioned studies and green tea’s antidepressant-like effects, research exploring green tea’s effect on BDNF would be justified. With BDNF being one of the most profound compounds studied in psychiatric disorders [25], it would be beneficial to identify nutritional interventions that impact its presence in the body. This highlights a gap in the current literature where further research is needed that explores how BDNF can be affected, if at all, by green tea and its bioactive constituents.

In addition to BDNF itself, the activation of its receptor, TrkB, plays a critical role in mediating its effects on neuronal growth, synaptic plasticity, and mood regulation. While none of the included RCTs assessed TrkB activity, animal studies have shown that exercise can enhance the BDNF/TrkB pathway [57], which may help explain why the single RCT in our review observed an upward trend in BDNF—potentially due to exercise rather than the GTE intervention.

As it pertains to mood disorders, they are not mutually exclusive from one another. Depression, anxiety, and sleep disorders have been found to share a two-way, bidirectional relationship with one another [58]. More specifically, over half of patients with depression also meet the diagnostic criteria for anxiety (49–81%), with the opposite being just as significant. Around 47–88% of those with anxiety also experience depression, highlighting the dynamic interplay mood disorders have with one another [59]. Sleep disorders seem to be no different. Some studies cite a high comorbidity rate between those with sleep disorders and depression, with the reverse also being true. Those with depression are more likely to experience sleep disorders [60]. As with depression, epidemiological findings show the development of sleep disorders in around 50% of people who have anxiety [61]. Nutritional psychiatry research needs to explore how nutritional interventions can impact even just one, thus potentially all three, of the major mood disorders.

Overall, these findings suggest that green tea extracts, along with bioactive compounds like L-theanine and EGCG, have beneficial effects on mood disturbance; more specifically, in reducing anxiety, depressive symptoms, and stress. Given the range in populations and dosages, more research is needed to explore optimal doses and formulations for a variety of populations.

### 4.4. Limitations

This review gives a comprehensive synthesis of randomized controlled trials exploring the impact of green tea on mood disorder symptomology and BDNF. A strength of this review was the inclusion of only randomized controlled trials, which ensured high-quality results while reducing confounding variables. The reliability of the findings is enhanced by the use of validated measurement tools and systematic screening protocols.

This has several limitations from the included studies. The studies varied in their methodologies, dosages, formulations, and intervention duration, which makes drawing definitive conclusions difficult. Utilizing subjective questionnaires for symptomology can also introduce bias. There is a lack of long-term intervention duration, which leaves the long-term benefits of these interventions unclear.

## 5. Conclusions

Overall, findings from this systematic review suggest that green tea, and its constituents—specifically L-theanine and EGCG) may offer beneficial effects on mood-related outcomes, including reductions in anxiety, stress, and depressive symptoms. Studies investigating L-theanine consistently reported reductions in anxiety and stress, particularly in clinical and subclinical populations. The effects of GTE and EGCG were varied, though improvements in mood metrics (e.g., STAI, SDS, MADRS, POMS, etc.) and depression scores were observed. Further research is needed to clarify the influence of variables like dose, supplement quality, and population characteristics on treatment efficacy. Importantly, although one study suggested a trend toward increased BDNF) the results were inconsistent and not statistically significant. These preliminary findings highlight the potential for green tea-based interventions as complementary strategies for mood regulation, though definitive conclusions cannot be drawn from individual studies alone. These results do, however, have important implications for the development and implementation of dietary interventions and supplements aimed at improving or treating mental health. Further high-quality randomized-controlled trials with larger samples, standardized interventions, and biomarker analyses like BDNF are needed to validate and confirm these results as well as to explore their potential underlying mechanisms.

### Future Directions and Clinical Implications

With the rise in mental health and mood disorders and people seeking alternative treatments for them, future research should prioritize high-quality RCTs with standardized dosing regimens and extended intervention durations to assess the long-term effectiveness of green tea. From a clinical perspective, the findings of this systematic review support the use of green tea as an evidence-based nutritional strategy for mental health and mood disorder symptomology. The use of green tea, GTE, EGCG, and L-theanine all shows promise in improving symptoms related to depression, stress, anxiety, and sleep. More research is needed to explore the impact of green tea and its bioactive compounds as they relate to serum BDNF.

## Figures and Tables

**Table 1:** PICOS Criteria for inclusion of studies.

Parameter	Criteria
Population	Adults over 18 years Healthy individuals and/or those with a diagnosis of mood disorders, including but not limited to, depression, anxiety, bipolar, stress, and sleep disorders Individuals with elevated levels of stress, anxiety, depression, or sleep disturbance at study screening
Intervention	Experimental studies with green tea or its bioactive compounds (i.e., EGCG, L-theanine, etc.) on mood and BDNF levels.
Comparison	Experimental studies with a placebo, control, or no intervention group
Outcome	Mood disorder symptomology using validated clinical scales and questionnaires and BDNF levels
Study Design	Randomized Controlled Trials

Abbreviations: EGCG, Epigallocatechin gallate; BDNF, Brain derived neurotropic factor.

## Data Availability

All data analyzed in this systematic review were extracted from previously published studies, which are cited throughout the manuscript. No new data were generated.

## Supplementary Materials

The following supporting information can be downloaded at: https://www.mdpi.com/article/10.3390/biomedicines13071656/s1. Supplementary documents provided include the PRISMA checklist.

## Abbreviations

The following abbreviations are used in this manuscript:

BDNF	Brain-derived neurotrophic factor
EGCG	Epigallocatechin gallate
GTE	Green tea extract
L-theanine	An amino acid found in tea
DSM-5	Diagnostic and Statistical Manual of Mental Disorders, Fifth Edition
CDC	Centers for Disease Control
PRISMA	Preferred Reporting Items for Systematic Reviews and Meta Analyses
PICOS	Population, Intervention, Comparison, Outcome, Study Design
STAI	State-Trait Anxiety Inventory
POMS	Profile of Mood States
HDRS	Hamilton Depression Rating Scale
VAS	Visual Analog Scale
PANSS	Positive and Negative Syndrome Scale
HAM-A	Hamilton Anxiety Rating Scale
MADRS	Montgomery-Asberg Depression Rating

## References

[1] Sekhon S., Gupta V. (2024). Mood Disorder. StatPearls [Internet].

[2] Spijker J., Claes S. (2014). Mood disorders in the DSM-5. Tijdschr. Psychiatr..

[3] Arnett J.J., Žukauskienė R., Sugimura K. (2014). The new life stage of emerging adulthood at ages 18–29 years: Implications for mental health. Lancet Psychiatry.

[4] (2024). *QuickStats:* Mental Health Treatment Trends Among Adults Aged ≥18 Years, by Age Group—United States, 2019–2023. MMWR Morb. Mortal. Wkly. Rep..

[5] Zimmerman M., Posternak M.A., Chelminski I. (2002). Symptom severity and exclusion from antidepressant efficacy trials. J. Clin. Psychopharmacol..

[6] Ivanov I., Schwartz J.M. (2021). Why Psychotropic Drugs Don’t Cure Mental Illness-But Should They?. Front. Psychiatry.

[7] Picardi A., Gaetano P. (2014). Psychotherapy of mood disorders. Clin. Pract. Epidemiol. Ment. Health.

[8] Fenn M.K., Byrne D.M. (2013). The key principles of cognitive behavioural therapy. InnovAiT: Educ. Inspir. Gen. Pract..

[9] Schaffler Y., Probst T., Jesser A., Humer E., Pieh C., Stippl P., Haid B., Schigl B. (2022). Perceived Barriers and Facilitators to Psychotherapy Utilisation and How They Relate to Patient’s Psychotherapeutic Goals. Healthcare.

[10] Graham H.N. (1992). Green tea composition, consumption, and polyphenol chemistry. Prev. Med..

[11] Yamamoto T., Juneja L.R., Chu D.-C., Kim M. (1997). Chemistry and Applications of Green Tea.

[12] Cabrera C., Artacho R., Giménez R. (2006). Beneficial Effects of Green Tea—A Review. J. Am. Coll. Nutr..

[13] Potenza M.A., Marasciulo F.L., Tarquinio M., Tiravanti E., Colantuono G., Federici A., Kim J.-A., Quon M.J., Montagnani M. (2007). EGCG, a green tea polyphenol, improves endothelial function and insulin sensitivity, reduces blood pressure, and protects against myocardial I/R injury in SHR. Am. J. Physiol. Endocrinol. Metab..

[14] Mokra D., Joskova M., Mokry J. (2022). Therapeutic Effects of Green Tea Polyphenol (−)-Epigallocatechin-3-Gallate (EGCG) in Relation to Molecular Pathways Controlling Inflammation, Oxidative Stress, and Apoptosis. Int. J. Mol. Sci..

[15] Talib W.H., Awajan D., Alqudah A., Alsawwaf R., Althunibat R., Abu AlRoos M., Al Safadi A., Abu Asab S., Hadi R.W., Al Kury L.T. (2024). Targeting Cancer Hallmarks with Epigallocatechin Gallate (EGCG): Mechanistic Basis and Therapeutic Targets. Molecules.

[16] Alam M., Gulzar M., Akhtar M.S., Rashid S., Zulfareen, Tanuja, Shamsi A., Hassan I. (2024). Epigallocatechin-3-gallate therapeutic potential in human diseases: Molecular mechanisms and clinical studies. Mol. Biomed..

[17] Onishi S., Meguro S., Pervin M., Kitazawa H., Yoto A., Ishino M., Shimba Y., Mochizuki Y., Miura S., Tokimitsu I. (2019). Green Tea Extracts Attenuate Brain Dysfunction in High-Fat-Diet-Fed SAMP8 Mice. Nutrients.

[18] Ko S., Jang W.S., Jeong J.-H., Ahn J.W., Kim Y.-H., Kim S., Chae H.K., Chung S. (2021). (−)-Gallocatechin gallate from green tea rescues cognitive impairment through restoring hippocampal silent synapses in post-menopausal depression. Sci. Rep..

[19] Baba Y., Inagaki S., Nakagawa S., Kobayashi M., Kaneko T., Takihara T. (2021). Effects of Daily Matcha and Caffeine Intake on Mild Acute Psychological Stress-Related Cognitive Function in Middle-Aged and Older Adults: A Randomized Placebo-Controlled Study. Nutrients.

[20] Kakuda T., Nozawa A., Sugimoto A., Niino H. (2002). Inhibition by theanine of binding of [^3^H]AMPA, [^3^H]kainate, and [^3^H]MDL 105,519 to glutamate receptors. Biosci. Biotechnol. Biochem..

[21] Moshfeghinia R., Sanaei E., Mostafavi S., Assadian K., Sanaei A., Ayano G. (2024). The effects of L-theanine supplementation on the outcomes of patients with mental disorders: A systematic review. BMC Psychiatry.

[22] Hidese S., Ota M., Wakabayashi C., Noda T., Ozawa H., Okubo T., Kunugi H. (2016). Effects of chronicl-theanine administration in patients with major depressive disorder: An open-label study. Acta Neuropsychiatr..

[23] Yokogoshi H., Kobayashi M., Mochizuki M., Terashima T. (1998). Effect of Theanine, r-Glutamylethylamide, on Brain Monoamines and Striatal Dopamine Release in Conscious Rats. Neurochem. Res..

[24] Ritsner M.S., Miodownik C., Ratner Y., Shleifer T., Mar M., Pintov L., Lerner V. (2010). L-theanine relieves positive, activation, and anxiety symptoms in patients with schizophrenia and schizoaffective disorder: An 8-week, randomized, double-blind, placebo-controlled, 2-center study. J. Clin. Psychiatry.

[25] Lin C.-C., Huang T.-L. (2020). Brain-derived neurotrophic factor and mental disorders. Biomed. J..

[26] Gliwińska A., Czubilińska-Łada J., Więckiewicz G., Świętochowska E., Badeński A., Dworak M., Szczepańska M. (2023). The Role of Brain-Derived Neurotrophic Factor (BDNF) in Diagnosis and Treatment of Epilepsy, Depression, Schizophrenia, Anorexia Nervosa and Alzheimer’s Disease as Highly Drug-Resistant Diseases: A Narrative Review. Brain Sci..

[27] Kulkarni S.K., Bhutani M.K., Bishnoi M. (2008). Antidepressant activity of curcumin: Involvement of serotonin and dopamine system. Psychopharmacology.

[28] Brunoni A.R., Lopes M., Fregni F. (2008). A systematic review and meta-analysis of clinical studies on major depression and BDNF levels: Implications for the role of neuroplasticity in depression. Int. J. Neuropsychopharmacol..

[29] Martinowich K., Manji H., Lu B. (2007). New insights into BDNF function in depression and anxiety. Nat. Neurosci..

[30] Castrén E., Monteggia L.M. (2021). Brain-Derived Neurotrophic Factor Signaling in Depression and Antidepressant Action. Biol. Psychiatry.

[31] A Siuciak J., Lewis D.R., Wiegand S.J., Lindsay R.M. (1997). Antidepressant-Like Effect of Brain-derived Neurotrophic Factor (BDNF). Pharmacol. Biochem. Behav..

[32] Huang E.J., Reichardt L.F. (2003). Trk Receptors: Roles in Neuronal Signal Transduction. Annu. Rev. Biochem..

[33] Jang S.-W., Liu X., Yepes M., Shepherd K.R., Miller G.W., Liu Y., Wilson W.D., Xiao G., Blanchi B., Sun Y.E. (2010). A selective TrkB agonist with potent neurotrophic activities by 7,8-dihydroxyflavone. Proc. Natl. Acad. Sci. USA.

[34] Saarelainen T., Hendolin P., Lucas G., Koponen E., Sairanen M., MacDonald E., Agerman K., Haapasalo A., Nawa H., Aloyz R. (2003). Activation of the TrkB Neurotrophin Receptor Is Induced by Antidepressant Drugs and Is Required for Antidepressant-Induced Behavioral Effects. J. Neurosci..

[35] Adachi M., Barrot M., Autry A.E., Theobald D., Monteggia L.M. (2008). Selective Loss of Brain-Derived Neurotrophic Factor in the Dentate Gyrus Attenuates Antidepressant Efficacy. Biol. Psychiatry.

[36] Page M.J., McKenzie J.E., Bossuyt P.M., Boutron I., Hoffmann T.C., Mulrow C.D., Shamseer L., Tetzlaff J.M., Akl E.A., Brennan S.E. (2021). The PRISMA 2020 statement: An updated guideline for reporting systematic reviews. BMJ.

[37] McGuinness L.A., Higgins J.P.T. (2021). Risk-of-bias VISualization (robvis): An R package and Shiny web app for visu-alizing risk-of-bias assessments. Res. Synth. Methods.

[38] Loftis J.M., Wilhelm C.J., Huckans M. (2012). Effect of epigallocatechin gallate supplementation in schizophrenia and bipolar disorder: An 8-week, randomized, double-blind, placebo-controlled study. Ther. Adv. Psychopharmacol..

[39] Ali D.M.S., Alireza M.S., Reza S.M., Jayran Z., SeyedAhmad S., Ali R.S., Saeid M.S., Ali A.-A. (2020). Effect of green tea consumption in treatment of mild to moderate depression in Iranian patients living with HIV: A double-blind randomized clinical trial. Chin. Herb. Med..

[40] Unno K., Furushima D., Tanaka Y., Tominaga T., Nakamura H., Yamada H., Taguchi K., Goda T., Nakamura Y. (2022). Improvement of Depressed Mood with Green Tea Intake. Nutrients.

[41] Unno K., Yamada H., Iguchi K., Ishida H., Iwao Y., Morita A., Nakamura Y. (2017). Anti-stress Effect of Green Tea with Lowered Caffeine on Humans: A Pilot Study. Biol. Pharm. Bull..

[42] Sadowska-Krępa E., Domaszewski P., Pokora I., Żebrowska A., Gdańska A., Podgórski T. (2019). Effects of medium-term green tea extract supplementation combined with CrossFit workout on blood antioxidant status and serum brain-derived neurotrophic factor in young men: A pilot study. J. Int. Soc. Sports Nutr..

[43] Yoto A., Murao S., Nakamura Y., Yokogoshi H. (2014). Intake of green tea inhibited increase of salivary chromogranin A after mental task stress loads. J. Physiol. Anthr..

[44] Lu K., Gray M.A., Oliver C., Liley D.T., Harrison B.J., Bartholomeusz C.F., Phan K.L., Nathan P.J. (2004). The acute effects of L-theanine in comparison with alprazolam on anticipatory anxiety in humans. Hum. Psychopharmacol. Clin. Exp..

[45] Unno K., Furushima D., Hamamoto S., Iguchi K., Yamada H., Morita A., Horie H., Nakamura Y. (2018). Stress-Reducing Function of Matcha Green Tea in Animal Experiments and Clinical Trials. Nutrients.

[46] Yoto A., Motoki M., Murao S., Yokogoshi H. (2012). Effects of L-theanine or caffeine intake on changes in blood pressure under physical and psychological stresses. J. Physiol. Anthr..

[47] Zhang Q., Yang H., Wang J., Li A., Zhang W., Cui X., Wang K. (2013). Effect of green tea on reward learning in healthy individuals: A randomized, double-blind, placebo-controlled pilot study. Nutr. J..

[48] Kimura K., Ozeki M., Juneja L.R., Ohira H. (2007). l-Theanine reduces psychological and physiological stress responses. Biol. Psychol..

[49] Hidese S., Ogawa S., Ota M., Ishida I., Yasukawa Z., Ozeki M., Kunugi H. (2019). Effects of L-Theanine Administration on Stress-Related Symptoms and Cognitive Functions in Healthy Adults: A Randomized Controlled Trial. Nutrients.

[50] Scholey A., Downey L.A., Ciorciari J., Pipingas A., Nolidin K., Finn M., Wines M., Catchlove S., Terrens A., Barlow E. (2012). Acute neurocognitive effects of epigallocatechin gallate (EGCG). Appetite.

[51] Peng B., Liu Z., Lin Y., Lin H., Huang J.A. (2014). The ameliorative effect of L-theanine on chronic unpredictable mild stress-induced depression in rats. J. Tea Sci..

[52] Shamabadi A., Fattollahzadeh-Noor S., Fallahpour B., Basti F.A., Ardakani M.-R.K., Akhondzadeh S. (2023). L-Theanine adjunct to risperidone in the treatment of chronic schizophrenia inpatients: A randomized, double-blind, placebo-controlled clinical trial. Psychopharmacology.

[53] Akbarialiabad H., Dahroud M.D., Khazaei M.M., Razmeh S., Zarshenas M.M. (2021). Green Tea, A Medicinal Food with Promising Neurological Benefits. Curr. Neuropharmacol..

[54] Hsieh M.-T., Lin C.-C., Lee C.-T., Huang T.-L. (2019). Abnormal Brain-Derived Neurotrophic Factor Exon IX Promoter Methylation, Protein, and mRNA Levels in Patients with Major Depressive Disorder. J. Clin. Med..

[55] Schröter K., Brum M., Brunkhorst-Kanaan N., Tole F., Ziegler C., Domschke K., Reif A., Kittel-Schneider S. (2019). Longitudinal multi-level biomarker analysis of BDNF in major depression and bipolar disorder. Eur. Arch. Psychiatry Clin. Neurosci..

[56] Martinotti G., Pettorruso M., De Berardis D., Varasano P.A., Pressanti G.L., De Remigis V., Valchera A., Ricci V., Di Nicola M., Janiri L. (2016). Agomelatine Increases BDNF Serum Levels in Depressed Patients in Correlation with the Improvement of Depressive Symptoms. Int. J. Neuropsychopharmacol..

[57] Cheng S.-M., Lee S.-D. (2022). Exercise Training Enhances BDNF/TrkB Signaling Pathway and Inhibits Apoptosis in Diabetic Cerebral Cortex. Int. J. Mol. Sci..

[58] Borges C., Ellis J.G., Marques D.R. (2023). The Role of Sleep Effort as a Mediator Between Anxiety and Depression. Psychol. Rep..

[59] Nguyen V.V., Zainal N.H., Newman M.G. (2022). Why Sleep is Key: Poor Sleep Quality is a Mechanism for the Bidirectional Relationship between Major Depressive Disorder and Generalized Anxiety Disorder Across 18 Years. J. Anxiety Disord..

[60] Han Z., Wang L., Zhu H., Tu Y., He P., Li B. (2024). Uncovering the effects and mechanisms of tea and its components on depression, anxiety, and sleep disorders: A comprehensive review. Food Res. Int..

[61] Chellappa S.L., Aeschbach D. (2022). Sleep and anxiety: From mechanisms to interventions. Sleep Med. Rev..

